# Amyloid β and Alzheimer’s Disease: Molecular Updates from Physiology to Pathology

**DOI:** 10.3390/ijms24097913

**Published:** 2023-04-26

**Authors:** Maria Laura Giuffrida

**Affiliations:** Institute of Crystallography, National Research Council (CNR-IC), 95126 Catania, Italy; marialaura.giuffrida@cnr.it; Tel.: +39-095-7338444

Alzheimer’s disease (AD) represents one of the most challenging disorders, and despite having been widely studied since its first identification, resolutive treatments are still far out of reach. The current knowledge of this progressive, incurable disease involves many aspects of its pathology, even those not closely related to the main hallmarks of AD, Amyloid-beta (Aβ) and Tau [1]. However, despite the emergence a new, complex perspective of AD as a multifactorial disease, the role of Aβ and Tau remains central. Looking at the current pipeline of AD drug development, great efforts are being made to move from symptomatic to disease-modifying treatments [2].

The ambitious goal to treat the pathology of AD cannot be achieved without a deep knowledge of the molecular mechanisms occurring in the earlier stages of the disease. To contribute to this issue, the aim of this Special Issue is to collect research that bridges the gap between the understanding of the contribution of Aβ in pathological conditions and the still unclarified involvement of this peptide in the physiological environment.

As such, the papers collected explore events from physiology to the occurrence of AD pathological conditions, providing new knowledge that is useful to prevent the transition from normal to dysfunctional neuronal metabolism and for the development of new therapeutic interventions.

Baidya A TK et al. [3] and Jeong YM et al. [4] both investigated the activities of Aβ in physiology. In the first paper, the authors provide new insight into cholinergic dysfunction, which is known to be central to the pathology of Alzheimer’s disease [5]. Using in silico analyses, they confirmed previous in vitro results showing that Aβ peptides at a physiological concentration can directly enhance the catalytic efficiency of choline acetyltransferase (ChAT). Molecular docking and dynamic analyses were conducted to further prove and clarify the mechanism of Aβ/ChAT interactions. The results contribute to a better comprehension of the native biological functions of Aβ peptides and their effect on the regulation of acetylcholine homeostasis.

Another important physiological process involved in the progression of AD has been reported by Jeong YM et al., whose results revealed a new mechanism of Aβ clearance mediated by brain lymphatic endothelial cells (BLECs). Impairments in the balance of production/degradation of amyloid peptides are considered one of the main processes responsible for sporadic forms of AD [6]. By using Zebrafish as in vivo model of pathology and a fluorescent-labeled Aβ1-42, the authors proved that BLECs cleared monomeric and oligomeric Aβ species differently. The more efficient degradation observed for Aβ monomers shed light on an important mechanism of Aβ regulation.

Another interesting and relevant issue has also been addressed by Bartley SC et al. [7]. In this work, starting with the assumption that Aβ can easily accumulate to form small aggregated species, the authors explored a potential functional effect of small Aβ oligomers (oAβs) during neurodevelopment. In the article, they elegantly showed that oAβs are normally present during the development of the embryonic retina, and their location and abundance are highly regulated as well as their contribution to retina histogenesis. These data could explain the evolutionarily conserved process of oligomerization and the gain-of-function activity shown by the peptide.

The loss of function and the gain of toxic function are very interesting issues in understanding the role played by Aβ in AD. One example of gain of toxic function is represented by the enhanced generation of Aβ in familial Alzheimer’s disease (FAD). Genetic mutations promote an over-expression of the peptide that progressively affects the balance between production and degradation, leading to an increase in the Aβ42/Aβ40 ratio, which is responsible for AD [8,9].

The study of Watanabe et al. [10] published in this Special Issue provides new insight into the activity of γ-secretase and the production of Aβ. Among the screened mutations that can affect γ-secretase cleavage, the authors found and investigated an Aph1 double mutant, Aph1aL L30F/T164A, that possesses protease activity without affecting its ability to form a complex with NCT and Pen2. The phenotype leads to an increase in the ε-cleavage of the enzyme.

To date, more than 150 mutations in PS1 have been associated with familial Alzheimer’s disease (FAD) [11,12]. A deeper knowledge of the related phenotypes might clarify the molecular mechanisms of Aβ toxicity with positive therapeutic implications for the disease.

Using different approaches, De Paula Faria et al. [13], Zambrano et al. [14], and Hoffmann et al. [15] aimed to identify new compounds or effective combined treatments to interfere with the progression of AD.

De Paula Faria’s work proposed the use of cannabidiol, already known to be neuroprotective and anti-inflammatory, in counteracting the glucose hypometabolism observed in AD brains.

Reduced glucose metabolism is one of the first signs observed in AD brains, which has been shown to contribute to cognitive decline over time. The recently identified commonalities between Alzheimer’s disease and type II diabetes have paved the way for new fields of research and therapeutic interventions [16]. The repositioning of drugs already approved for type II diabetes, or the study of new molecules able to restore glucose metabolism, represent new hope in Alzheimer’s therapy.

In this interesting study, the role of cannabidiol on brain glucose impairments induced by streptozotocin (STZ) injection in rats was investigated. By comparing behavioral tests and [18F]FDG PET images on whole brains, they proved that cannabidiol treatment could represent an important therapeutic option for Alzheimer’s disease, protecting against the cognitive decline induced by glucose hypometabolism.

Zambrano et al. provide insight into the protective effect of AV CRI104P4 on Aβ toxicity. The compound is a donepezil-huprine hybrid that possesses multitarget functions, such as a strong inhibitory activity of human acetylcholinesterase and Aβ aggregation in vitro and in vivo as well as a proven ability to improve short-term memory in APPSL transgenic mice. In the study, the authors focused their attention on the underlying mechanism of cell membrane protection of AV CRI104P4 against Aβ toxicity.

X-ray diffraction, differential scanning calorimetry (DSC), and scanning electron microscopy (SEM) have been used to identify the mechanisms by which the compound protects the membranes of human erythrocytes or molecular models of their outer and inner membranes, such as dimyristoyl phosphatidylcholine (DMPC) and dimyristoyl phosphatidyl ethanolamine (DMPE), from Aβ insult.

Another compelling study included in this issue, with the aim of identifying a disease-modifying therapy, is the study of Hoffmann et al. The authors investigated the combined effect of Glutamyl Cyclase Inhibitor PQ912 (Varoglutamstat) and the Murine Monoclonal Antibody P8D-C06 (m6) on the formation and clearance of pGlu-Aβ in transgenic mice brains.

It has been reported that N-terminally truncated and pyroglutamate-modified Abeta (AβpE3) peptides are abundantly present in the brain of AD patients. The conversion of pGlu-Aβ glutamic acid into pyroglutamic acid catalyzed by glutaminyl cyclase (QC) leads to an increase in hydrophobicity, which in turn accelerates the formation of highly neurotoxic oligomers [17,18]. Additionally, pGlu-modified Aβ is more resistant to degradation, causing accumulation in AD brains [19]. For this reason, targeting pyroglutamate-modified peptides by the use of anti-pGlu3-Aβ antibody m6 (murine PBD-C06) and preventing their formation using the glutaminyl cyclase inhibitor, Varoglutamstat (PQ912), might represent a good strategy to address the progression of Alzheimer’s disease. In addition, the synergistic drug combinations proposed by the authors maintain therapeutic efficacy, allowing for reductions in each compound, thus overcoming the toxicity and side effects associated with high dosages.

Overall, the articles collected in this Special Issue represent an interesting contribution to the advancement of knowledge about Alzheimer’s disease and the role of amyloid beta within and beyond the disease. The critical view emerging from the collection could be beneficial for the refinement of existing pharmacological interventions and/or the design of new drug candidates.
